# Enhancing the Quality of Ginseng–Astragalus Medicinal Food Using Twin-Screw Extrusion

**DOI:** 10.3390/foods14162886

**Published:** 2025-08-20

**Authors:** Yuankuo Sun, Tie Jin, Guanhao Li

**Affiliations:** 1 College of Agricultural, Yanbian University, Yanji 133002, China; 2056074133k@gmail.com; 2 Key Innovation Laboratory for Deep and Intensive Processing of Yanbian High Quality Beef, Ministry of Agriculture and Rural Affairs, Yanji 133000, China

**Keywords:** ginseng, astragalus, twin-screw extrusion, chemical properties, antioxidant properties, flavor, bacteriostatic effects

## Abstract

Twin-screw extrusion is a promising method to transform medicinal herbs into functional food ingredients. However, limited research has focused on the application of this technique to ginseng–astragalus compounds. In this study, the response surface methodology (RSM) was used to optimize the extrusion process (screw speed, temperature, and moisture content). The optimal parameters (208 rpm, 128 °C, 29%) significantly increased ginsenoside, polysaccharide, phenolic content, and antioxidant capacities (DPPH•, OH•, O_2_^−^•). Furthermore, extrusion improved the aroma profile while reducing bitterness, as revealed by electronic nose and electronic tongue, and PCA. The treated product also exhibited enhanced antibacterial activity. These findings demonstrate that twin-screw extrusion is an effective tool for developing medicinal food products with improved quality and biofunctionality. The response surface methodology model showed high reliability (R^2^ > 0.95) with prediction errors and relative standard deviations below 5%, confirming the robustness of the optimization.

## 1. Introduction

Panax ginseng is a widely recognized medicinal and edible plant with a long history of use in Asia spanning over 2000 years [1,2]. It is rich in nutrients and pharmacologically active compounds, including ginsenosides, polysaccharides, phenolic compounds, peptides, vitamins, and essential oils [3,4]. Ginsenosides are the primary bioactive constituents of ginseng, classified into diol-type and triol-type forms, with Rg3 being a rare saponin notable for its anticancer potential [5,6].

Astragalus membranaceus contains saponins, flavonoids such as calycosin-7-glucoside, and other phytochemicals with recognized bioactivities [7,8,9]. In traditional medicine, these two herbs are often co-formulated to enhance shared benefits such as immune modulation, antioxidant protection, and overall vitality, while minimizing side effects.

However, conventional processing methods such as drying, slicing, grinding, or solvent extraction often suffer from limitations, including poor extraction efficiency of active compounds and suboptimal sensory qualities. Twin-screw extrusion, as a modern and sustainable food processing technology, offers a solution by enabling thermal–mechanical treatment that modifies the structural, nutritional, and functional properties of food matrices [10,11]. The high temperature, pressure, and shear forces generated during extrusion can disrupt plant cell walls, enhance the release of bioactive compounds, and trigger physicochemical changes such as the Maillard reaction, thereby improving the functionality and palatability of herbal products [12].

Previous studies have highlighted the potential of extrusion in improving the bioactivity of plant-based ingredients. Martín-Diana et al. demonstrated enhanced phenolic content and antioxidant activity in teff-rice extrudates [13], while Gui et al. and Zhang et al. reported increases in ginsenosides and antioxidant activity in extruded red ginseng products [14,15]. Despite these findings, there remains a notable research gap in the application of extrusion for the food-grade processing of ginseng–astragalus mixtures. Most research to date has focused on pharmacological analysis or chemical extraction, rather than scalable processing methods compatible with functional food development. Moreover, comprehensive evaluations encompassing antioxidant activity, compound transformation, flavor modulation, and antibacterial properties after extrusion remain limited.

However, most existing studies on the extrusion of herbal materials, such as ginseng and astragalus, have primarily focused on single-factor optimization or basic compositional changes, without integrating comprehensive bioactive profiling, antioxidant evaluation, and flavor characterization under systematically optimized processing conditions. Moreover, the specific mechanisms by which extrusion influences ginsenoside transformation and flavor compound development remain underexplored. This study addresses these gaps by applying response surface methodology to optimize twin-screw extrusion parameters for a ginseng–astragalus functional blend, followed by a multi-parameter assessment of bioactive retention, antioxidant activities, and sensory attributes. The findings provide novel insights into process–quality relationships and offer a scalable approach for developing high-value herbal functional foods.

## 2. Experimental Section

### 2.1. Materials

Ginseng and Astragalus membranaceus roots were purchased from Yanji City, Jilin Province, China. Sulfuric acid, methanol, aluminum nitrate, anhydrous ethanol, sodium hydroxide, phenol, rutin, gallic acid, sodium nitrite, sodium carbonate, and standard reagents, including ginsenoside Rb1, Rc, Rd, Rg3, Re, CK, and calycosin-7-glucoside, were obtained from Shanghai Macklin Biochemical Co., Ltd. (Shanghai, China). All reagents were of analytical grade.

### 2.2. Preparation of Ginseng–Astragalus Compound Powder

The raw herbs were pulverized using a high-speed grinder (DFT-50; Purchased from Shandong Shengrun Machinery Co., Jinan, China) and sieved through a 60-mesh screen. Ginseng and astragalus powders were mixed at a 1:1 (*w*/*w*) ratio and adjusted to various moisture levels (23–31%) by adding deionized water. The mixtures were sealed in polyethylene bags and equilibrated at 60 °C for 24 h prior to extrusion.

### 2.3. Twin-Screw Extrusion Process

Extrusion was performed using a laboratory twin-screw extruder (DSE-30; Shandong Shengrun Machinery Co., Jinan, China) with a screw diameter of 32 mm and screw length 742 mm. The feed rate was maintained at 150 g/min. The selected parameter ranges for screw speed (150–250 rpm), barrel temperature (110–140 °C), and feed moisture (23–31%) were determined based on preliminary trials conducted to ensure smooth material conveyance, prevent blockage, and achieve adequate thermal–mechanical treatment without excessive degradation of bioactives. These ranges are also consistent with extrusion conditions reported in previous studies on herbal and cereal-based functional foods, which demonstrated optimal compound retention and product texture within similar operating windows [16]. Extrusion parameters were optimized using response surface methodology (RSM) via Design-Expert 13.0 software (Stat-Ease Inc., Minneapolis, MN, USA).

After extrusion, samples were dried at 80 °C for 6 h in a convection oven, ground through an 80-mesh sieve, and sealed for storage. One gram of extrudate was extracted with 30 mL of 70% ethanol in a water bath at 70 °C for 45 min, followed by 60 min of ultrasonic extraction and centrifugation at 4000 rpm for 30 min. The supernatant was collected for further analysis. Table 1 presents the factor levels for the RSM, and Table 2 lists the experimental design.

### 2.4. Determination of Bioactive Components

The chemical and antioxidant properties of the control ginseng–astragalus compound and the seventeen extruded ginseng–astragalus compounds after extrusion. The total ginsenoside content of the samples was determined by Soxhlet extraction, vanillin-sulfuric acid color development, and ultraviolet-visible spectrophotometry [17].

Appropriate amounts of ginsenoside Re standard, ginsenoside Rb1, Rc, Rd, Rg3, CK standard, and Calycosin-7-glucoside standard were taken, respectively, and the control solution was obtained with different mass concentrations by 70% methanol. The separation was performed on a ZORBAX SB-C18 column (250 mm × 4.6 mm, 5 μm) at a controlled flow rate of 1 mL/min with an injection volume of 20 μL. For the determination of ginsenosides, the detection wavelength of the instrument was set at 203 nm, and the column temperature was 30 °C; for the determination of Calycosin-7-glucoside, the detection wavelength of the instrument was set at 260 nm, and the column temperature was 25 °C. The gradient elution was performed with acetonitrile as the mobile phase A and water as the mobile phase B. The separation was carried out in a gradient elution mode. The HPLC detection was performed according to the elution procedures in Table 3, Table 4 and Table 5.

Total polysaccharides were quantified by the phenol–sulfuric acid method [18], total flavonoids using rutin as the reference standard [19], and total phenolics using the Folin–Ciocalteu assay [20].

### 2.5. Antioxidant Activity Assays

DPPH radical scavenging activity, ABTS radical scavenging activity, hydroxyl radical scavenging rate, and superoxide anion radical scavenging rate were determined according to the test procedure of the kit manual. DPPH•, ABTS•^+^, hydroxyl radical (•OH), and superoxide anion radical (O_2_^−^•) assay kits were purchased from Nanjing Jiancheng Bioengineering Institute (Nanjing, China).

### 2.6. Determination of Optimal Operating Parameters and Validation Tests

A multi-response optimization was conducted using Design-Expert software to maximize chemical composition and antioxidant activity. The optimized extrusion parameters were validated through confirmatory experiments to assess prediction accuracy and process stability.

### 2.7. Flavor Profile Analysis

Flavor volatiles were assessed using an electronic nose (PEN 3, Airsense Analytics, Schwerin, Germany). Measurements followed ASTM E253-09 standards [21]. PCA analysis was conducted using WinMuster software v1.6.2.

Using the Japanese INSENT taste analysis system for electronic, the analysis was performed electronically using the INSENT taste analysis system from Japan. The system was calibrated according to ISO 13302:2003 [22], and the data were normalized and subjected to LDA and PCA for dimensional reduction.

### 2.8. Measurement of Bacteriostatic Effect

The inhibition zones were measured by the disc diffusion method (Kirby–Bauer), using *E. coli*, *Staphylococcus aureus*, and *Salmonella typhimurium*. Samples were prepared in 0.1% DMSO. The concentration of the bacteria in the culture medium was adjusted to be 10^5^~10^6^ cfu/mL. Three sets of soaked filter paper sheets were placed on the surface of the medium. The Petri dishes were inverted and placed into the 37 °C constant temperature oven for 12 h. The size of the diameter of the inhibition circle was measured and concluded. The maximum dilution of the sample solution that could prevent the growth of bacteria was defined as its minimum inhibitory concentration [23].

### 2.9. Statistical Analysis

All RSM experimental runs were conducted as independent replicates on separate batches of material (n = 3 for each treatment), ensuring biological replication rather than pseudo-replication. Data were analyzed using Microsoft Excel 2023, SPSS 26.0 (IBM Corp., Armonk, NY, USA), and Design-Expert 13.0 software. E-nose and e-tongue data were processed using the instrument’s proprietary software with PCA and LDA analysis. A significance level of *p* < 0.05 was applied for all statistical tests.

## 3. Results and Discussion

### 3.1. Effect of Twin-Screw Extrusion on Bioactive Components

Twin-screw extrusion significantly enhanced the bioactive compound content in ginseng–astragalus compounds. The statistical details of each experimental run, including raw values and coded factor levels, are summarized in Tables S1 and S2 (provided in Supplementary Material for conciseness). These data complement the 3D response surface plots presented here by providing the underlying numerical results used for model fitting. As shown in Table S1 (Supplementary Material), the total ginsenoside content increased from 18.52 mg/g in the unprocessed mixture to 22.06–28.66 mg/g after extrusion. This increase may be attributed to the disruption of plant cell walls under high shear, temperature, and pressure, facilitating the release and transformation of saponins. The influence of process variables on total ginsenoside content followed the order: barrel temperature > screw speed > material moisture content. Statistical analysis revealed that barrel temperature (B) and its interaction with screw speed (AB) had highly significant effects (*p* < 0.01).

Interestingly, triol-type ginsenosides such as Re decreased post-extrusion, while diol-type ginsenosides (Rb1, Rc, Rd, and Rg3) increased, suggesting partial structural transformation. The increase in Rg3 content may result from the cleavage of C-20 glycosyl residues and hydroxyl isomerization, a pathway typical of red ginseng transformation [24]. The high shear force breaks the plant cell wall, causing the saponins to be released from the plant matrix. Water and other solvents in the material may act as solvents under high temperatures and pressure, helping to promote the dissolution and recrystallization of saponins, thus affecting the ratio of different saponin types. At the same time, high temperature and pressure will accelerate the hydrolysis and transformation process of saponins and promote the isomerization or rearrangement reaction of ginsenosides, leading to the transformation of the originally existing triol-type ginsenosides to the diol type. Specifically, the combined thermal–mechanical energy during extrusion can disrupt the C-O glycosidic bonds at the C-3 or C-20 positions of ginsenosides, leading to the stepwise removal of sugar moieties (deglycosylation). Under high shear, localized temperature spikes and mechanical friction promote partial hydrolysis and isomerization, facilitating the conversion of triol-type ginsenosides (e.g., Re) into diol-type forms (e.g., Rg3, CK) through dehydration and rearrangement reactions.

In terms of polysaccharides and phenolics (Supplementary Table S2), extrusion increased total polysaccharides from 136.78 mg/g to 143.76–153.06 mg/g, and total phenolics from 5.20 mg/g to 5.02–6.26 mg/g. The enhancement is likely due to mechanical degradation of polysaccharide chains and enhanced release of phenolic compounds and glucose compounds upon cell disruption [25,26,27]. The most influential factor for polysaccharides was screw speed, whereas moisture content most affected phenolic yield. Interaction terms AC and AB were statistically significant (*p* < 0.01 or *p* < 0.05). Total flavonoids showed a decline post-extrusion (from 1.04 mg/g to 0.69–0.93 mg/g), possibly due to thermal degradation. However, the major isoflavone in Astragalus membranaceus: calycosin-7-glucoside increased significantly (*p* < 0.05), indicating selective conversion or stabilization of certain flavonoid compounds under extrusion conditions.

### 3.2. Antioxidant Properties

**Figure 1:**
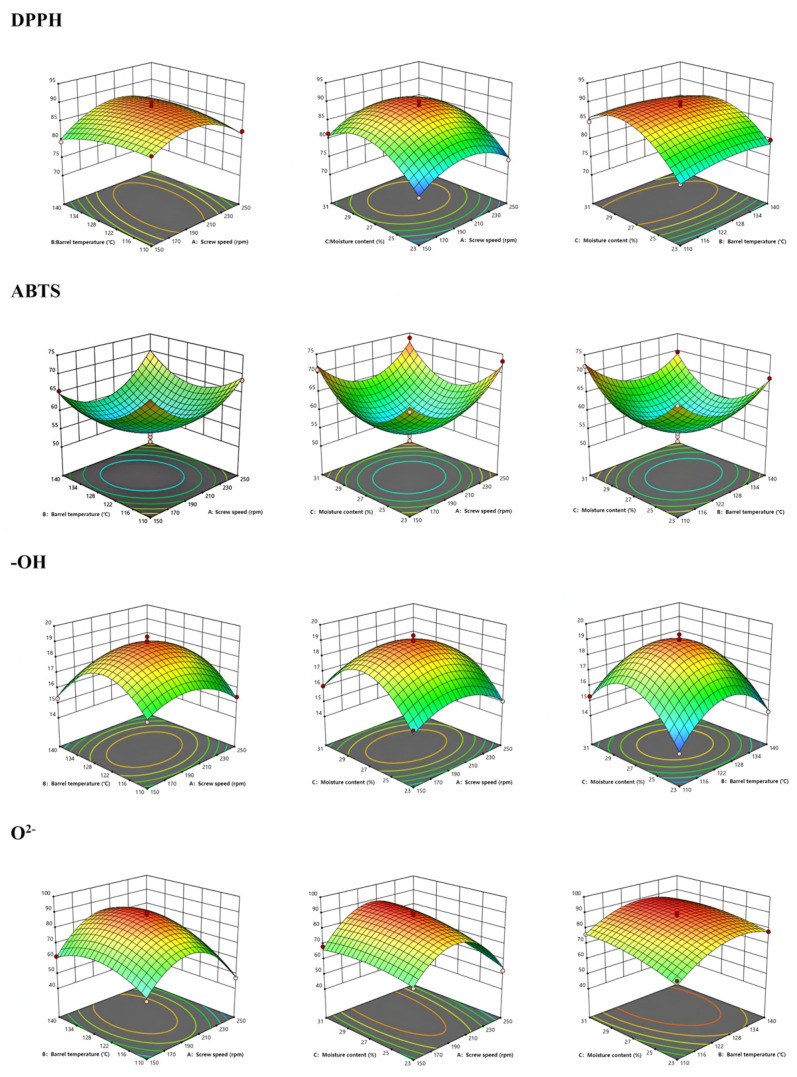
Effect of extrusion operation parameters on DPPH radical scavenging, ABTS radical, hydroxyl radical scavenging, and superoxide anion radical scavenging in ginseng astragalus extrudates.

The antioxidant capacity of the extruded samples improved notably (Supplementary Table S2, Figure 1). Antioxidant activity improved significantly following extrusion. Scavenging activities for DPPH•, hydroxyl radical (•OH), and superoxide anion radical (O_2_-•) were elevated, consistent with the increased levels of saponins, polysaccharides, and phenolics. Interestingly, ABTS•^+^ radical scavenging activity slightly declined after extrusion, in contrast to the increases observed for DPPH•, hydroxyl radical (•OH), and superoxide anion radical (O_2_^−^•) scavenging. This discrepancy may be attributed to the different reaction mechanisms and compound selectivity of the assays. The ABTS•^+^ assay responds to hydrophilic antioxidants, whereas DPPH• primarily reflects lipophilic radical scavengers, and hydroxyl/superoxide assays target highly reactive oxygen species. Extrusion may selectively degrade certain ABTS-reactive hydrophilic antioxidants (e.g., vitamin C, some phenolic acids) due to thermal and oxidative stress, while enhancing the release or transformation of lipophilic compounds such as ginsenosides and flavonoid aglycones, which are more effective in the other assays. In addition, the formation of Maillard reaction products with strong DPPH• and O_2_^−^• scavenging capacity but lower ABTS•^+^ reactivity could further contribute to the observed trend. The order of influence on antioxidant indices varied by assay:

(a) DPPH•: moisture content > screw speed > temperature.
(b) ABTS•^+^: temperature > screw speed > moisture.
(c) Hydroxyl (•OH): moisture > temperature > screw speed.
(d) Superoxide (O_2_^−^•): temperature > moisture > screw speed.

Significant interaction effects (e.g., AB, AC) were observed across several assays (*p* < 0.05 or *p* < 0.01), validating the complex interplay between extrusion variables and antioxidant activity [28,29].

### 3.3. Optimization and Model Validation

RSM analysis using Design-Expert 13 identified the optimal extrusion parameters as: screw speed 208 rpm, barrel temperature 128 °C, and moisture content 29%. Model validation (Table 6, Table 7 and Table 8) showed that prediction errors and RSDs were <5%, indicating robust model reliability for industrial-scale application.

### 3.4. Flavor Profile and Odor Analysis

**Figure 2:**
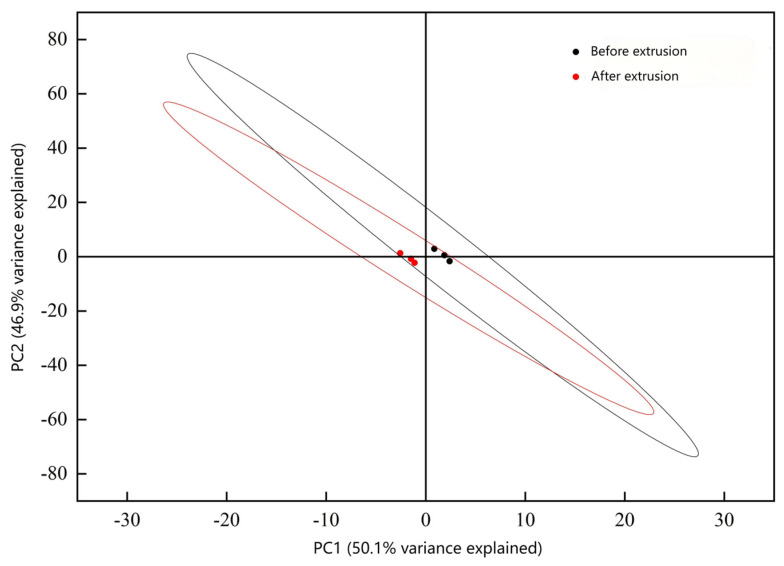
Principal component analysis of electronic tongue.

**Figure 3:**
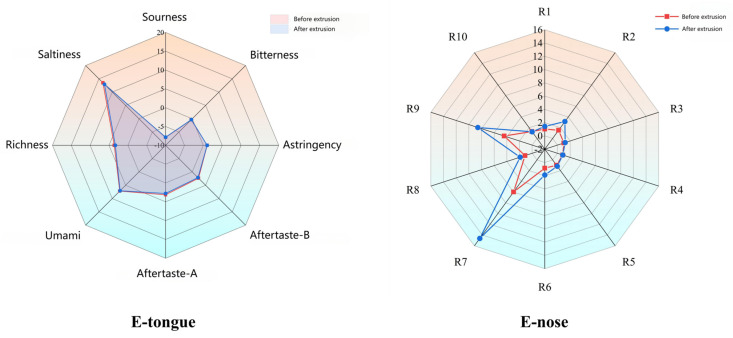
Radar charts of electronic tongue response value and electronic nose response value.

**Figure 4:**
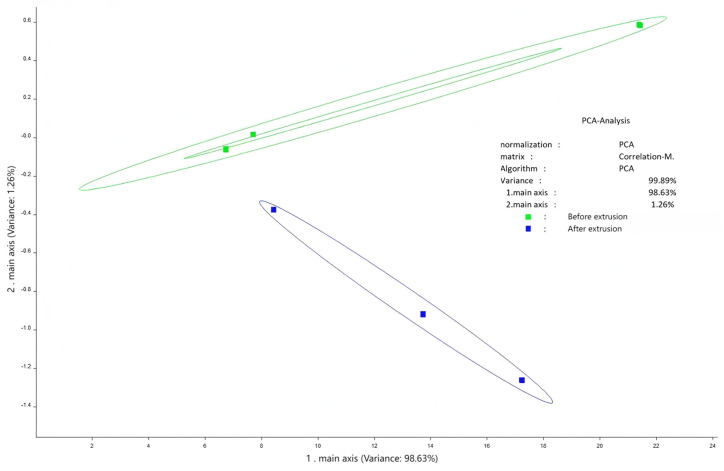
Electronic nose principal component analysis diagram.

**Figure 5:**
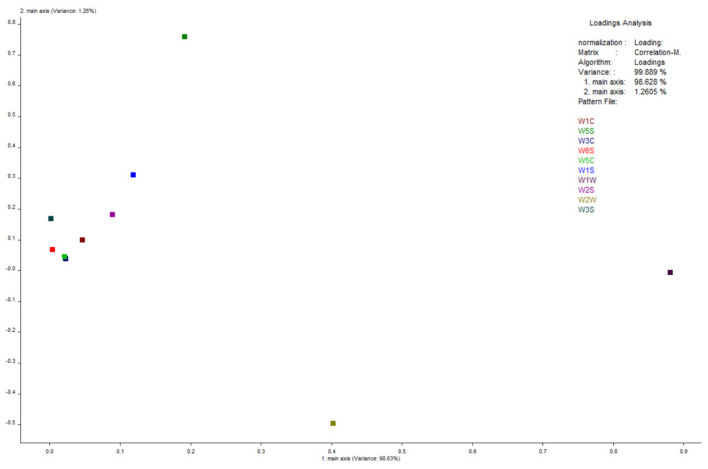
Electronic nose loading analysis diagram.

**Figure 6:**
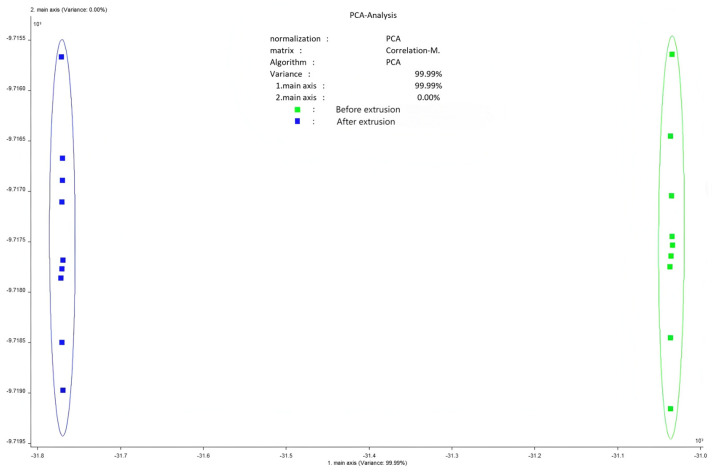
LDA analysis diagram of electronic nose.

Flavor analysis using electronic tongue and nose technologies showed distinct changes post-extrusion (Figure 2, Figure 3, Figure 4, Figure 5 and Figure 6). PCA results confirmed clear separability of samples before and after extrusion (≥99.8% cumulative contribution). In terms of taste profile, trends mirrored those observed in thermally processed broccoli stems [30], where bitterness and umami showed significant shifts, and PCA differentiation was over 99%. Taste indicators such as bitterness and astringency were reduced, and aroma intensity, especially for volatile classes like sulfides, terpenes, and nitrogen oxides, was enhanced.

Similar to studies in pea flour [31], our E-nose PCA analysis revealed clear separation between pre-extrusion and post-extrusion samples, with increased signals in sulfide- and terpene-sensitive sensors (W1W, W2W), indicative of aroma intensification.

### 3.5. Antibacterial Activity

**Figure 7:**
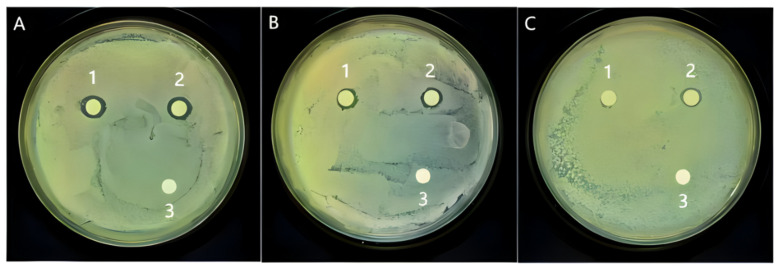
Measurement results of antibacterial zone diameter: (**A**): *E. coli*; (**B**): *S.aureus*; (**C**): *Salmonella typhi*; 1: Ginseng–Astragalus Compound Group; 2: the Ginseng–Astragalus Extruded Compound Group; 3: Blank.

Antibacterial assays demonstrated that extruded samples had improved inhibitory effects against *E. coli*, *S. aureus*, and *Salmonella typhi* (Table 9, Figure 7). Inhibition zones increased slightly, and MIC values were halved for some pathogens (8→4 mg/mL for *E. coli*). The enhanced antimicrobial activity is attributed to increased concentrations of bioactives such as Rg3 and calycosin-7-glucoside, consistent with previous findings (Table 10) [32].

## 4. Conclusions

In this study, a functional ginseng–astragalus compound was successfully developed using twin-screw extrusion technology. Response surface methodology (RSM) was employed to optimize the processing parameters, with the optimal conditions identified as a screw speed of 208 rpm, barrel temperature of 128 °C, and feed moisture content of 29%.

Under these conditions, significant improvements in total ginsenosides, polysaccharides, and specific bioactives such as Rg3 and calycosin-7-glucoside were observed. Correspondingly, antioxidant and antibacterial activities were enhanced, and sensory quality, flavor, and aroma were notably improved.

The optimized twin-screw extrusion process demonstrates strong potential for scale-up in the production of commercial herbal functional foods, given its continuous operation, controllable parameters, and compatibility with existing food processing lines. However, certain limitations should be acknowledged, including the potential loss of highly volatile aroma compounds during high-temperature extrusion and the relatively high energy input required for sustained operation. Future work should explore process refinements, such as lower-temperature post-extrusion treatments or aroma recovery systems, to maximize product quality while maintaining industrial feasibility.

## Figures and Tables

**Table 1:** Response surface level values.

Grouping	Factor A (rpm)	Factor B (°C)	Factor C (%)
−1	150	110	23
0	200	125	27
1	250	140	31

**Table 2:** Experimental protocol for the design of response surface centered assemblies.

Grouping	Factor A (rpm)	Factor B (°C)	Factor C (%)
1	150	110	27
2	250	110	27
3	150	140	27
4	250	140	27
5	150	125	23
6	250	125	23
7	150	125	31
8	250	125	31
9	200	110	23
10	200	140	23
11	200	110	31
12	200	140	31
13	200	125	27
14	200	125	27
15	200	125	27
16	200	125	27
17	200	125	27

**Table 3:** Mobile phase elution procedure 1.

Time (min)	Mobile Phase A (%)	Mobile Phase B (%)
0~35	19	81
35~50	19~29	81~71
50~70	29	71
70~80	29~35	71~65
80~100	35~40	65~60

**Table 4:** Mobile phase elution procedure 2.

Time (min)	Mobile Phase A (%)	Mobile Phase B (%)
0~15	25	75
15~45	25~40	75~60
45~50	40	60
50~80	40~62	60~38

**Table 5:** Mobile phase elution procedure 3.

Time (min)	Mobile Phase A (%)	Mobile Phase B (%)
0~20	10~30	90~70
20~25	30	70
25~35	30~60	70~40
35~45	60~10	40~90

**Table 6:** Constraints on the optimization of response surface parameters.

Factors	Goal	Lower Limit	Upper Limit	Lower Limit of a Unit	Upper Limit of a Unit	Importance
Factor A (rpm)	Is in range	150	250	1	1	3
Factor B (°C)	Is in range	110	140	1	1	3
Factor C (%)	Is in range	23	31	1	1	3
Ginseng total saponin (mg/g)	Maximize	22.06	28.66	1	1	3
Polysaccharide (mg/g)	Maximize	143.764	153.057	1	1	3
Total flavonoids (mg/g)	Maximize	0.7009	0.9415	1	1	3
Total phenol (mg/g)	Maximize	5.0197	6.2586	1	1	3
Re (mg/g)	Maximize	5.3	6.01	1	1	3
Rb1 (mg/g)	Maximize	4.74	6.24	1	1	3
Rc (mg/g)	Maximize	1.09	1.53	1	1	3
Rd (mg/g)	Maximize	0.79	1.14	1	1	3
Rg3 (mg/g)	Maximize	0.11	0.18	1	1	3
CK (mg/g)	Maximize	0.08	0.1	1	1	3
Calycosin-7-glucoside (mg/g)	Maximize	0.46	0.69	1	1	3
DPPH (%)	Maximize	73.9	90.143	1	1	3
ABTS (%)	Maximize	51.487	73.593	1	1	3
−OH (%)	Maximize	14.0448	19.3286	1	1	3
O^2−^ (%)	Maximize	47.1092	90.005	1	1	3

**Table 7:** Verification test results of optimum machining parameters.

Testing Indicators	Optimized Data	Triple-Parallel Data			Results	Inaccuracies	Standard Deviation	RSD
Ginseng total saponin (mg/g)	28.17	28.24	28.1	27.96	18.1	0.25%	0.14	0.5%
Polysaccharide (mg/g)	151.108	151.19	148.95	150.66	148.79	1.1%	1.037	0.69%
Total flavonoids (mg/g)	0.853	0.844	0.838	0.846	0.843	1.23%	0.004	0.49%
Total Phenol (mg/g)	6.136	6.015	6.134	6.139	6.096	0.66%	0.07	1.15%
Re (mg/g)	5.593	5.585	5.581	5.597	5.588	0.1%	0.008	0.15%
Rb1 (mg/g)	5.872	5.813	5.84	5.844	5.832	0.68%	0.017	0.29%
Rc (mg/g)	1.377	1.357	1.379	1.374	1.37	0.51%	0.012	0.84%
Rd (mg/g)	1.01	0.99	0.98	0.99	0.987	2.36%	0.006	0.59%
Rg3 (mg/g)	0.173	0.17	0.168	0.171	0.17	1.96%	0.002	0.9%
CK (mg/g)	0.09	0.09	0.08	0.09	0.087	3.85%	0.006	6.66%
Calycosin-7-glucoside (mg/g)	0.56	0.571	0.579	0.588	0.579	3.34%	0.009	1.47%
DPPH (%)	88.648	88.607	88.612	88.613	88.611	0.04%	0.003	0%
ABTS (%)	58.427	58.487	58.454	58.451	58.464	0.06%	0.02	0.03%
-OH (%)	18.255	18.178	18.206	18.216	18.2	0.3%	0.02	0.11%
O^2−^ (%)	89.518	89.534	89.546	89.543	89.541	0.03%	0.006	0.01%
Mean error and mean RSD						1.1%		0.93%

**Table 8:** Test results of sensors of electronic tongue.

Testing Program	Grouping	
	Ginseng and Astragalus Compound	Ginseng and Astragalus Compound
Sourness	−7.830 ± 0.4817	−7.903 ± 0.4603
Bitterness	−0.3233 ± 0.1301	−0.3933 ± 0.1201
Astringency	1.027 ± 0.03512	1.017 ± 0.02309
Aftertaste-B	2.347 ± 0.07371	2.150 ± 0.03606 *
Aftertaste-A	3.15 ± 0.03606	2.797 ± 0.01528 **
Umami	7.193 ± 0.03055	7.080 ± 0.03606 *
Richness	3.607 ± 0.3053	3.327 ± 0.2501
Saltiness	13.49 ± 0.114	12.94 ± 0.07024 *

Note: ** indicates highly significant difference (*p* < 0.01), * indicates more significant difference (*p* < 0.05).

**Table 9:** Circle of inhibition diameters before and after extrusion of ginseng–astragalus compound.

Strains	Culture Medium	Inhibitory Circle Diameter (mm)	
		Pre-Extrusion	Post-Extrusion
*E.coli*	Beef Paste Peptone Agar Medium	9.3 ± 0.06 ^b^	9.8 ± 0.07 ^a^
*S.aureus*	Beef Paste Peptone Agar Medium	8.2 ± 0.09 ^b^	8.5 ± 0.01 ^a^
*Salmonella typhi*	Beef Paste Peptone Agar Medium	7.4 ± 0.05 ^b^	8.4 ± 0.02 ^a^

Note: Mean ± standard deviation (n = 3), and completely different shoulder-script letters indicate significant differences (*p* < 0.05).

**Table 10:** MIC of ginseng–astragalus complex before and after extrusion.

Concentration of Sample Solution (mg/mL)	Pre-Extrusion			Post-Extrusion		
	*E. coli*	*S. aureus*	*Salmonella Typhi*	*E. coli*	*S. aureus*	*Salmonella Typhi*
32	−	−	−	−	−	−
16	−	−	+	−	−	−
8	−	+	+	−	−	−
4	+	+	+	−	+	+
2	+	+	+	+	+	+
1	+	+	+	+	+	+
0.5	+	+	+	+	+	+
Positive control	+	+	+	+	+	+
Negative control	−	−	−	−	−	−

Note: + indicates bacterial growth; − indicates no bacterial growth.

## Data Availability

The raw data supporting the conclusions of this article will be made available by the authors on request.

## Supplementary Materials

The following supporting information can be downloaded at: https://www.mdpi.com/article/10.3390/foods14162886/s1. Table S1: Experimental design and responses for ginsenoside content, polysaccharides, and antioxidant activities. Table S2: Coded and actual factor levels for each run and model fitting results.
