# Supplementary material for: Enhancing the Quality of Ginseng–Astragalus Medicinal Food Using Twin-Screw Extrusion
Source: Foods. 2025 Aug 20;14(16):2886. doi: 10.3390/foods14162886 (PMC12385580; doi:10.3390/foods14162886)
Supplement: Supplementary file 1 [file foods-14-02886-s001.zip › foods-3804858-supplementary.pdf]

Table S1. Saponin content.

**Grouping**	**Ginseng total saponin (mg/g)**	**Triol Ginsenoside**	**Diol Ginsenosides**					**Astragaloside**
		**Re (mg/g)**	**Rb1 (mg/g)**	**Rc (mg/g)**	**Rd (mg/g)**	**Rg3 (mg/g)**	**CK (mg/g)**	**Calycosin-7-glucoside (mg/g)**
Control group	18.52±0.30^{a}	7.51±0.17^{e}	4.87±0.15^{ab}	0.91±010^{a}	0.84±0.02^{ab}	0.08±0.01^{a}	0.09±0.01	0.44±0.03^{a}
1	22.59±0.34^{bc}	5.68±0.21^{abcd}	5.71±0.17^{ef}	1.09±0.07^{ab}	0.98±0.12^{bcde}	0.13±0.02^{bc}	0.09±0.01	0.46±0.03^{ab}
2	22.06±0.63^{b}	5.45±0.40^{abc}	4.9±0.21^{ab}	1.21±0.07^{bcde}	0.91±0.12^{abcd}	0.13±0.01^{bc}	0.08±0.00	0.51±0.03^{abcd}
3	23.52±0.66^{cd}	5.56±0.15^{abcd}	5.47±0.12^{de}	1.19±0.05^{bcde}	0.99±0.10^{bcde}	0.11±0.02^{b}	0.09±0.01	0.53±0.03^{abcde}
4	24.56±0.76^{e}	6.01±0.21^{d}	5.40±0.20^{de}	1.15±0.10^{bcd}	0.85±0.06^{abc}	0.11±0.02^{b}	0.09±0.01	0.57±0.03^{cdefg}
5	23.89±0.32^{de}	5.49±0.30^{abc}	5.96±0.15^{fgh}	1.19±0.06^{bcde}	0.88±0.06^{abc}	0.12±0.01^{b}	0.09±0.01	0.54±0.03^{abcdef}
6	24.03±0.07^{de}	5.56±0.30^{abcd}	5.35±0.10^{cd}	1.28±0.08^{cdef}	0.80±0.06^{a}	0.15±0.02^{cdef}	0.10±0.01	0.56±0.03^{bcdefg}
7	23.85±0.68^{de}	5.67±0.26^{abcd}	4.74±0.10^{a}	1.27±0.10^{bcdef}	0.79±0.06^{a}	0.15±0.01^{cde}	0.09±0.00	0.50±0.05^{abc}
8	24.40±0.49^{de}	5.72±0.21^{abcd}	4.98±0.15^{ab}	1.19±0.07^{bcde}	0.79±0.05^{a}	0.16±0.01^{def}	0.09±0.01	0.53±0.03^{abcde}
9	24.61±0.14^{e}	5.85±0.21^{bcd}	5.34±0.10^{cd}	1.33±0.12^{def}	0.97±0.15^{bcd}	0.12±0.01^{b}	0.09±0.00	0.62±0.05^{efgh}
10	25.66±0.07^{f}	5.34±0.26^{ab}	5.57±0.12^{de}	1.16±0.10^{bcd}	0.92±0.08^{abcd}	0.13±0.01^{bcd}	0.09±0.01	0.6±0.08^{defgh}
11	24.59±0.12^{e}	5.80±0.35^{abcd}	5.89±0.10^{fg}	1.10±0.06^{bc}	0.88±0.00^{abc}	0.13±0.02^{bcd}	0.09±0.01	0.55±0.10^{bcdefg}
12	25.70±0.06^{f}	5.91±0.30^{cd}	5.07±0.10^{bc}	1.22±0.15^{bcde}	1.01±0.05^{cde}	0.17±0.01^{ef}	0.08±0.01	0.52±0.03^{abcde}
13	28.33±0.07^{g}	5.50±0.15^{abc}	6.11±0.21^{gh}	1.30±0.12^{def}	1.07±0.06^{de}	0.16±0.01^{ef}	0.09±0.02	0.55±0.08^{bcdef}
14	28.48±0.81^{g}	5.60±0.21^{abcd}	6.10±0.15^{gh}	1.53±0.12^{g}	0.98±0.01^{bcde}	0.18±0.02^{f}	0.09±0.00	0.51±0.03^{abcd}
15	28.49±0.81^{g}	5.30±0.25^{a}	6.20±0.20^{gh}	1.35±0.06^{efg}	1.01±0.06^{cde}	0.18±0.02^{f}	0.10±0.00	0.63±0.04^{fgh}
16	28.45±0.06^{g}	5.60±0.20^{abcd}	6.24±0.15^{h}	1.45±0.12^{fg}	1.14±0.12^{e}	0.16±0.02^{ef}	0.09±0.00	0.69±0.08^{h}
17	28.66±0.07^{g}	5.38±0.17^{ab}	6.07±0.15^{gh}	1.43±0.06^{fg}	1.06±0.12^{de}	0.16±0.01^{def}	0.09±0.01	0.65±0.05^{gh}

Note: Data with the same letter on the shoulder in the same column indicate non-significant differences ($P > 0.05$), and completely different letters on the shoulder indicate significant differences ($P < 0.05$).

Table S2. Chemical and antioxidant properties.

Grouping	Polysaccharide (mg/g)	Total flavonoids (mg/g)	Total Phenol (mg/g)	DPPH (%)	ABTS (%)	-OH (%)	O_2- (%)
Control group	136.78 ± 2.48^{a}	1.04 ± 0.06^{e}	5.20 ± 0.07^{abc}	60.40 ± 1.2^{a}	78.34 ± 1.15^{h}	11.13 ± 1.21^{a}	53.77 ± 1.49^{b}
1	145.30 ± 0.85^{ab}	0.83 ± 0.00^{bc}	5.02 ± 0.18^{a}	84.45 ± 1.98^{efghi}	72.87 ± 1.06^{efg}	16.08 ± 3.48^{ab}	55.55 ± 2.74^{b}
2	146.07 ± 2.43^{abc}	0.83 ± 0.08^{bc}	5.50 ± 0.15^{abcde}	82.31 ± 2.32^{def}	68.36 ± 1.04^{cd}	15.41 ± 2.18^{ab}	47.11 ± 1.31^{a}
3	144.94 ± 2.10^{ab}	0.76 ± 0.01^{ab}	5.49 ± 0.15^{abcde}	79.40 ± 1.78^{cd}	65.45 ± 2.41^{c}	15.31 ± 0.40^{ab}	61.50 ± 1.29^{c}
4	148.92 ± 8.14^{bcd}	0.78 ± 0.04^{bc}	5.05 ± 0.04^{a}	83.67 ± 1.44^{defgh}	68.63 ± 0.95^{cde}	15.80 ± 1.55^{ab}	66.17 ± 0.70^{cde}
5	148.25 ± 6.64^{bcd}	0.84 ± 0.03^{c}	5.52 ± 0.47^{abcde}	73.90 ± 0.65^{b}	67.95 ± 0.65^{cd}	15.51 ± 2.41^{ab}	63.68 ± 1.94^{cd}
6	149.11 ± 0.37^{bcd}	0.85 ± 0.05^{c}	5.67 ± 0.13^{bcdef}	74.12 ± 4.51^{b}	73.13 ± 2.27^{fg}	15.04 ± 4.07^{ab}	52.35 ± 3.03^{ab}
7	143.76 ± 0.91^{ab}	0.84 ± 0.02^{c}	5.68 ± 0.21^{cdefg}	81.39 ± 2.13^{cdef}	70.90 ± 1.93^{defg}	16.05 ± 1.85^{ab}	68.39 ± 3.25^{de}
8	149.54 ± 4.14^{bcd}	0.93 ± 0.04^{d}	5.99 ± 0.47^{efg}	82.00 ± 1.24^{cdef}	73.60 ± 4.31^{g}	15.27 ± 4.23^{ab}	70.09 ± 3.88^{e}
9	148.83 ± 1.05^{bcd}	0.82 ± 0.03^{bc}	5.92 ± 0.17^{efg}	77.36 ± 0.25^{bc}	69.55 ± 3.45^{cdefg}	14.05 ± 2.96^{ab}	67.91 ± 1.69^{de}
10	148.52 ± 3.65^{bcd}	0.69 ± 0.01^{a}	5.10 ± 0.13^{ab}	79.75 ± 6.2^{cde}	68.80 ± 1.73^{cdef}	14.26 ± 2.24^{ab}	77.92 ± 2.20^{f}
11	144.70 ± 2.39^{ab}	0.81 ± 0.02^{bc}	5.25 ± 0.07^{abcd}	84.73 ± 1.49^{fghi}	71.98 ± 4.19^{defg}	15.29 ± 3.08^{ab}	76.17 ± 2.45^{f}
12	145.96 ± 0.39^{abc}	0.82 ± 0.03^{bc}	5.97 ± 0.52^{efg}	83.34 ± 0.78^{defg}	69.61 ± 1.24^{cdefg}	14.63 ± 1.23^{ab}	80.80 ± 4.07^{f}
13	151.70 ± 1.20^{cd}	0.80 ± 0.01^{bc}	6.16 ± 0.36^{fg}	88.04 ± 2.62^{ghij}	51.49 ± 0.40^{a}	18.97 ± 1.53^{b}	89.54 ± 5.10^{g}
14	151.83 ± 0.95^{cd}	0.82 ± 0.03^{bc}	6.26 ± 0.15^{g}	88.15 ± 0.32^{hij}	52.81 ± 3.07^{a}	18.56 ± 3.21^{b}	86.64 ± 1.51^{g}
15	151.92 ± 2.36^{cd}	0.83 ± 0.06^{bc}	6.01 ± 0.47^{efg}	88.82 ± 1.36^{ij}	58.82 ± 1.13^{b}	19.33 ± 1.36^{b}	88.17 ± 2.24^{g}
16	153.06 ± 1.34^{d}	0.82 ± 0.03^{bc}	6.17 ± 0.28^{fg}	89.07 ± 1.11^{ij}	59.32 ± 0.77^{b}	19.04 ± 2.65^{b}	86.19 ± 2.33^{g}
17	151.94 ± 1.25^{cd}	0.83 ± 0.05^{bc}	5.79 ± 0.32^{defg}	90.14 ± 0.67^{j}	53.69 ± 0.65^{a}	18.65 ± 2.13^{b}	90.01 ± 0.67^{g}

Note: Data with the same letter on the shoulder in the same column indicate non-significant differences ($P > 0.05$), and completely different letters on the shoulder indicate significant differences ($P < 0.05$).
